# Association of an evidence-informed nasogastric enteral nutrition nursing pathway with gastrointestinal recovery and nutritional status in neurosurgical critical care: a retrospective quasi-experimental cohort study

**DOI:** 10.3389/fnut.2026.1883367

**Published:** 2026-07-20

**Authors:** Feng Sun, Wenjun Shan, Huihui Zhang, Keqing Yan, Yu Ding

**Affiliations:** Department of Neurosurgery, The Affiliated Suqian Hospital of Xuzhou Medical University (Nanjing Drum Tower Hospital Group Suqian Hospital), Suqian, Jiangsu, China

**Keywords:** enteral nutrition, evidence-informed nursing, feeding intolerance, nasogastric tube, neurosurgical intensive care, quasi-experimental study

## Abstract

**Background:**

Critically ill neurosurgical patients frequently require nasogastric enteral nutrition (EN), yet impaired consciousness, dysphagia, ventilation, and gastrointestinal dysmotility predispose to underfeeding and intolerance. We evaluated whether implementation of an evidence-informed nasogastric EN nursing pathway was associated with improved gastrointestinal recovery and nutritional status in routine neurosurgical critical care.

**Methods:**

This single-center retrospective quasi-experimental cohort study reviewed consecutive adults requiring nasogastric EN in a neurosurgical intensive care unit between January 2022 and January 2024. Patients managed before pathway implementation formed the usual-care cohort (*n* = 56) and those managed afterward the evidence-informed pathway cohort (*n* = 56); a defined implementation transition interval was excluded a priori. Primary outcomes were time to initial EN tolerance, first documented flatus, and first defecation. Secondary outcomes were 7-day feeding compliance, 14-day feeding intolerance, and serial nutritional biomarkers. Effect estimates are reported with 95% confidence intervals (CIs).

**Results:**

Baseline characteristics were balanced. Compared with usual care, the pathway cohort had shorter time to initial EN tolerance (mean difference, −1.30 days; 95% CI, −1.88 to −0.72), first documented flatus (−1.60 days; 95% CI, −1.88 to −1.32), and first defecation (−1.60 days; 95% CI, −2.04 to −1.16; all *P* < 0.001). Seven-day feeding compliance was higher (92.86% versus 69.64%; risk difference, 23.2 percentage points; 95% CI, 9.4–37.0; *P* = 0.002), and 14-day feeding intolerance was lower (10.71% versus 28.57%; risk difference, −17.9 percentage points; 95% CI, −32.2 to −3.5; *P* = 0.032). Hemoglobin, albumin, and prealbumin increased in both cohorts, with greater gains in the pathway cohort and significant group-by-time interactions (*P* < 0.001). Documented adherence to the core pathway elements was high, with a composite adherence of 87.5%.

**Conclusion:**

The evidence-informed nasogastric EN nursing pathway was associated with faster documented gastrointestinal recovery, higher feeding compliance, fewer intolerance events, and more favorable nutritional biomarker trajectories. Because the design was nonrandomized, findings are associative and warrant prospective multicenter confirmation.

## Introduction

Critically ill neurosurgical patients are particularly vulnerable to nutritional deterioration because acute brain injury and the physiological burden of neurosurgical illness frequently coincide with impaired consciousness, dysphagia, reduced airway protection, mechanical ventilation, autonomic dysfunction, and prolonged immobility. The metabolic response to severe neurologic injury is not uniform across the trajectory of critical illness. During the early acute phase, inflammation, endocrine stress, sedation, vasoactive therapy, and evolving organ dysfunction may alter energy expenditure and gastrointestinal motility, whereas during the subsequent recovery phase persistent protein catabolism may compromise immune competence, wound healing, respiratory muscle strength, and neurologic rehabilitation. Patients who cannot eat orally therefore require a structured approach to nutrition support that is both physiologically appropriate and operationally reliable. Enteral nutrition is generally preferred over parenteral nutrition when the gastrointestinal tract is functional and feeding can be delivered safely, because it supports gut integrity, contributes to mucosal and immune barrier function, and avoids several catheter-associated complications of parenteral delivery (1–3). In neurosurgical critical care, however, the practical delivery of enteral nutrition is frequently difficult. Reduced consciousness and swallowing impairment commonly necessitate nasogastric tube feeding rather than oral intake, while delayed gastric emptying, vomiting, aspiration risk, abdominal distension, diarrhea, frequent diagnostic or operative interruptions, and uncertainty about gastric residual volume can produce repeated feeding holds and cumulative energy and protein deficits. For these reasons, the success of enteral nutrition depends not only on the physician prescription but also on the nursing procedures that govern tube placement verification, securement, positioning, infusion advancement, tolerance surveillance, documentation, and timely escalation. Prior work in neurocritical and general intensive care has emphasized early enteral nutrition, protocolized feeding advancement, and prevention of nasogastric tube-related adverse events (4–6). Nasogastric tube misplacement remains an important and preventable safety concern that nursing leadership and standardized verification can mitigate (7), and structured transition feeding protocols have improved nutritional delivery in acute neurosurgical populations (8). Nevertheless, the literature remains limited regarding real-world implementation of nursing-led, evidence-informed enteral nutrition pathways within neurosurgical intensive care units. Usual care often contains evidence-based elements but is less standardized and more dependent on individual bedside experience, which increases practice variation and may delay recognition of feeding intolerance, an outcome whose definitions and pathophysiology have been progressively clarified (9). A pathway that integrates contemporary evidence with the local workflow may reduce variation, improve feeding delivery, and detect intolerance earlier. Because the comparison in the present study reflects a unit-level change in routine nursing practice rather than patient-level randomization, a retrospective quasi-experimental cohort framework is the appropriate design, permitting clinically meaningful assessment of implementation while explicitly acknowledging the risks of temporal confounding and residual bias. We therefore conducted a retrospective quasi-experimental cohort analysis of critically ill neurosurgical patients who required nasogastric enteral nutrition before and after implementation of an evidence-informed nursing pathway. The primary aim was to determine whether the pathway was associated with faster documented gastrointestinal recovery. The secondary aims were to compare feeding compliance, feeding intolerance, and serial nutritional biomarkers during the first 14 days of nasogastric enteral nutrition support.

## Materials and methods

### Study design and setting

**FIGURE 1:**
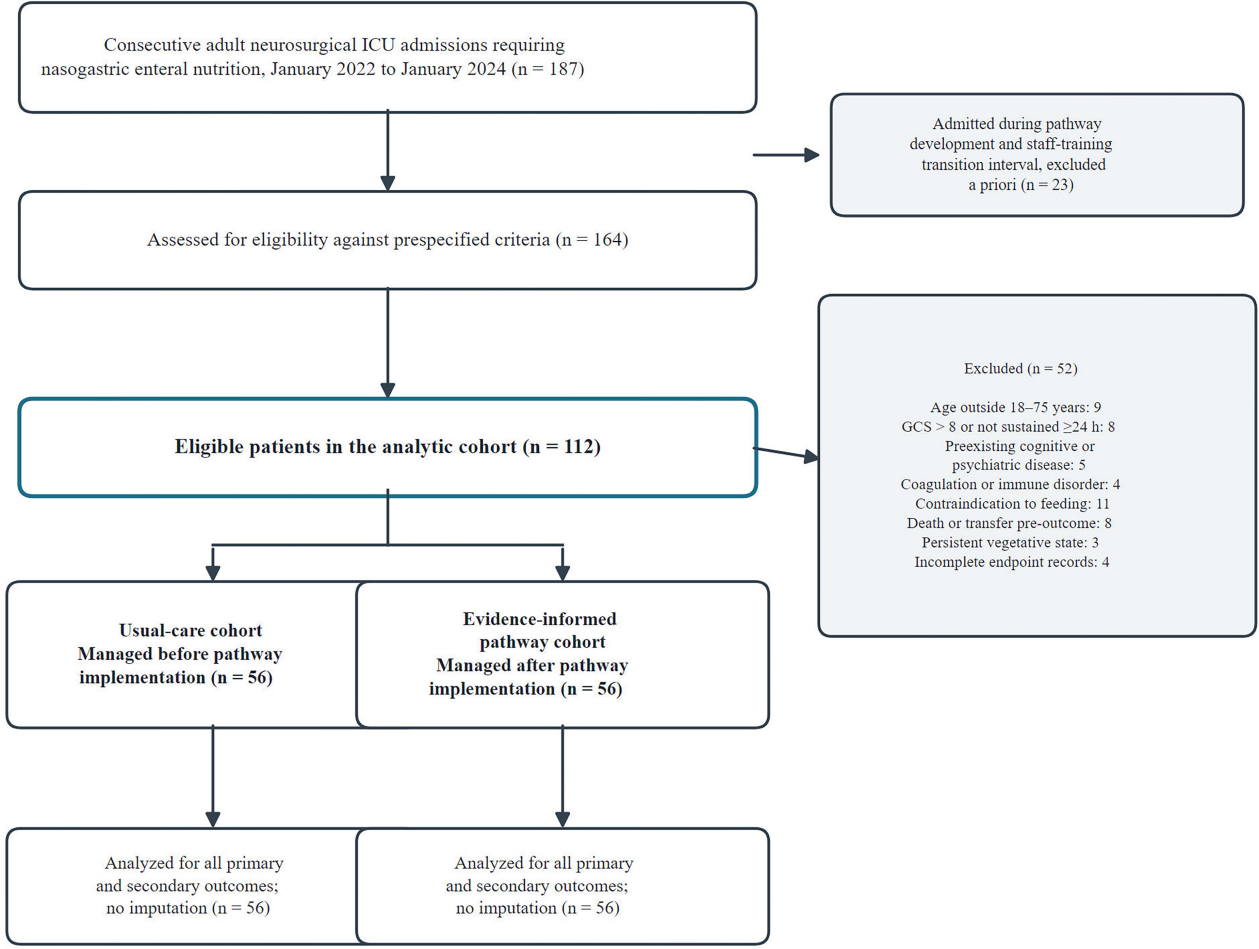
Study flow diagram. Screening, a priori exclusion of patients admitted during the implementation transition interval, eligibility exclusions with reasons, and final allocation to the usual-care and evidence-informed pathway cohorts, prepared in accordance with STROBE recommendations. The implementation transition interval denotes the period during which the pathway was developed and nursing staff were trained, which was excluded a priori from exposure classification to limit contamination between care models. Abbreviations: EN, enteral nutrition; GCS, Glasgow Coma Scale; ICU, intensive care unit; STROBE, Strengthening the Reporting of Observational Studies in Epidemiology.

This single-center retrospective quasi-experimental cohort study was conducted in the neurosurgical intensive care unit of The Affiliated Suqian Hospital of Xuzhou Medical University. Routinely collected clinical records from January 2022 through January 2024 were used to compare outcomes before and after implementation of a standardized evidence-informed nasogastric enteral nutrition (EN) nursing pathway. Patients treated during the pre-implementation period constituted the usual-care cohort, and patients treated after implementation constituted the evidence-informed pathway cohort. Because the exposure was determined by a unit-level practice change rather than patient-level assignment, patients were not randomized, and no allocation procedure was used. Bedside nurses and treating clinicians could not be blinded because the pathway was embedded in routine nursing workflow. To limit contamination between care models, the interval during which the pathway was developed and nursing staff were trained was designated a priori as an implementation transition interval and was excluded from exposure classification. Reporting followed the Strengthening the Reporting of Observational Studies in Epidemiology (STROBE) recommendations for observational cohort studies (10). The flow of participants through screening, exclusion, and analysis is summarized in Figure 1.

### Participants

Eligible patients were adults 18–75 years of age admitted to the neurosurgical intensive care unit with acute severe neurosurgical brain injury confirmed by computed tomography or magnetic resonance imaging, admission within 24 h of disease onset, a Glasgow Coma Scale (GCS) score of 3–8 sustained for at least 24 h, and a documented clinical requirement for nasogastric EN. The diagnostic spectrum comprised basal ganglia hemorrhage, ruptured intracranial aneurysm with hemorrhage, and massive cerebral infarction requiring intensive neurosurgical management. This broad neurosurgical definition was adopted to align eligibility with the diagnoses actually represented in the cohort and to avoid misclassification as isolated traumatic brain injury. Patients were excluded for severe preexisting cognitive or psychiatric disease precluding baseline assessment, major coagulation or immune disorders judged to materially alter the clinical course, contraindications to enteral feeding such as bowel ischemia, active gastrointestinal bleeding, or intestinal obstruction or perforation requiring withholding of EN, death or transfer before outcome assessment, persistent vegetative state during the observation period, or incomplete records for primary endpoints. Intestinal obstruction or perforation was treated as a contraindication to feeding rather than to nasogastric tube placement itself.

### Exposure groups and nursing pathways

The usual-care cohort received the institutional standard of nasogastric EN nursing used before pathway implementation, which included assessment of clinical condition, vital-sign monitoring, nasogastric tube insertion and verification according to institutional policy, routine tube fixation, head-of-bed elevation when feasible, initiation of feeding at approximately 25 mL per hour when ordered, and advancement based on bedside tolerance assessment and physician direction. Usual care was not characterized as non-evidence-based; rather, it represented standard practice without a unified written pathway, formal competency auditing, predefined intolerance algorithm, or structured feeding-compliance review.

The evidence-informed pathway was developed by a multidisciplinary group of neurosurgical physicians, the head nurse, and neurosurgical intensive care nurses, who reviewed relevant guidelines, consensus statements, institutional safety policies, and published literature on EN in critical illness, neurocritical care feeding, nasogastric tube safety, aspiration prevention, and feeding intolerance. Before implementation, nursing staff received structured training in tube safety, aspiration-risk reduction, feeding advancement, intolerance recognition, documentation, and escalation. The pathway specified prefeeding risk assessment, confirmation of the EN indication, review of gastrointestinal contraindications, standardized nasogastric tube securement, head-of-bed elevation to 30– 45 degrees unless contraindicated, regular oral care, verification of tube position before feeding and after suspected displacement, and documented assessment of abdominal findings, emesis, stool pattern, respiratory symptoms, and gastric residual volume when clinically indicated by local policy. Feeding was initiated at a low rate and advanced gradually toward the prescribed target when no intolerance criteria were present. The same institutional EN formulary was used in both periods, and caloric and protein goals were individualized by the treating team according to body weight, neurologic severity, organ function, and metabolic risk. When intolerance occurred, the pathway required prompt bedside reassessment, temporary rate reduction or feeding hold when clinically appropriate, repeat tube-position evaluation when displacement was suspected, physician notification, consideration of prokinetic therapy or formula adjustment, and documented re-escalation once symptoms resolved. A structured comparison of the two care models is provided in Supplementary Table S1.

Energy and protein targets were established for every patient at the initiation of nasogastric enteral nutrition and were derived in the same manner in both periods. Indirect calorimetry was not routinely available in the unit; targets were therefore calculated from weight-based predictive equations consistent with contemporary critical-care nutrition guidance, namely an energy goal of approximately 25 kcal per kilogram of actual body weight per day and a protein goal of 1.2–2.0 g per kilogram per day, with adjustment for neurologic severity, organ function, and metabolic risk (1–3). Feeding was initiated at 20–25 mL per hour and, in the absence of any intolerance criterion, advanced by approximately 20 mL per hour every 8–12 h toward the individualized goal rate, such that the prescribed target was ordinarily achieved within 3–5 days. Rate advancement was permitted only when no intolerance criterion was present; when intolerance occurred, the infusion was reduced to the last tolerated rate or briefly held and was subsequently re-escalated once the precipitating symptoms resolved. The principal procedural distinction between the cohorts was that the pathway specified these initiation, advancement, hold, and re-escalation thresholds explicitly and required their documentation, whereas usual care advanced feeding according to bedside judgment without a unified written algorithm. Seven-day feeding compliance was defined against this individualized prescription as the proportion of patients who received at least 90% of the cumulative prescribed enteral volume by day 7.

### Outcomes and definitions

The primary outcomes were gastrointestinal recovery measures recorded after nasogastric tube placement: time to initial EN tolerance, time to first clinically documented flatus, and time to first defecation. Initial EN tolerance was defined as the ability to receive continuous nasogastric EN at or above 30 mL per hour for 24 h without vomiting, aspiration concern, clinically significant abdominal distension, or gastric retention requiring feeding interruption. First flatus was defined as the first passage of gas documented by bedside nursing assessment or clinical observation; because most patients had severe impairment of consciousness, this endpoint was treated as a pragmatic clinical documentation outcome rather than an exact physiological timestamp. First defecation was defined as the first recorded bowel movement after tube placement. Secondary outcomes were 7-day feeding compliance, 14-day feeding intolerance, and nutritional biomarker trajectories. Feeding compliance was defined as achievement of at least 90% of the individualized prescribed EN volume by day 7. Feeding intolerance during the 14-day observation period comprised gastric retention (gastric residual volume greater than 200 mL at scheduled assessment or a residual-volume finding leading to feeding interruption), nausea or vomiting, aspiration (new coughing, respiratory difficulty, oxygenation deterioration, or nutrient-like airway secretions temporally associated with feeding), diarrhea (more than three watery stools per day or output meeting institutional criteria), and abdominal distension (visible swelling with reduced bowel sounds, absent flatus, or discomfort when assessable). Nutritional biomarkers were hemoglobin, albumin, prealbumin, triglycerides, and total cholesterol, measured before tube placement and on days 7 and 14 after placement. The outcome framework was prespecified before data abstraction. Gastrointestinal recovery measures were designated primary because they are mechanistically proximal, objectively recorded, and captured early in the feeding course, whereas feeding compliance, feeding intolerance, and biomarker trajectories were designated secondary delivery and biochemical endpoints. We acknowledge that time to first flatus and time to first defecation are motility-dependent measures that can be influenced by sedation, fluid balance, mechanical ventilation, concomitant gastrointestinal-active medications, and the enteral formula; these endpoints are therefore interpreted as supportive indicators of gastrointestinal recovery rather than as isolated effects of the nursing pathway. The clinically actionable delivery endpoint, 7-day feeding compliance, is reported alongside them so that the proximal motility measures and the delivery measure can be appraised together.

### Data collection and quality control

Demographic characteristics, primary diagnosis, GCS score, Acute Physiology and Chronic Health Evaluation II (APACHE II) score, feeding data, intolerance events, concomitant gastrointestinal-active medications (acid-suppressive agents and prokinetic agents), vasoactive or catecholamine support, continuous sedation, mechanical ventilation, and laboratory values were abstracted from the electronic medical record and nursing documentation. Two trained reviewers independently verified the extracted dataset against source records, and discrepancies were resolved by discussion with a senior investigator. The final analytic dataset included only patients with complete data for primary endpoints and prespecified biomarker time points, and no statistical imputation was performed. Because albumin and prealbumin are influenced by inflammation, fluid status, hepatic synthesis, and exogenous albumin administration, they were interpreted as supportive biochemical markers rather than stand-alone diagnostic measures of malnutrition (11). For the pathway cohort, adherence to the intervention was assessed by auditing the nursing record for documented completion of the core bundle elements, comprising structured prefeeding risk assessment, standardized tube securement and verification, head-of-bed elevation to 30 –45 degrees, scheduled tolerance surveillance, application of the predefined intolerance algorithm when a trigger was present, and the days 3–7 feeding-compliance review. Element-level adherence and a composite measure reflecting completion of all core elements were summarized descriptively and are reported in Supplementary Table S2.

### Sample-size considerations

The sample comprised all eligible patients identified during the predefined study window, and no formal a priori sample-size calculation was performed because the analysis used existing consecutive records. In a post hoc evaluation, 56 patients per cohort provided approximately 90% power to detect a between-cohort difference of 1.0 day in time to initial EN tolerance, assuming a standard deviation of 1.6 days and a two-sided alpha of 0.05. Analyses of infrequent intolerance subcategories were therefore considered descriptive and hypothesis-generating.

### Statistical analysis

Analyses were performed with SPSS version 27.0 (IBM Corporation, Armonk, NY, United States). Continuous variables were assessed for distributional assumptions using histograms, Q-Q plots, and the Shapiro-Wilk test; normally distributed variables are presented as mean ± standard deviation and were compared using independent-samples *t*-tests. Categorical variables are presented as number and percentage and were compared using the χ^2^ test with continuity correction or the Fisher exact test, as appropriate. Repeated laboratory measurements were analyzed using repeated-measures analysis of variance with group, time, and group-by-time interaction terms, with Bonferroni adjustment for pairwise comparisons. Effect estimates are reported as mean differences for continuous outcomes and absolute risk differences for categorical outcomes, each with 95% confidence intervals (CIs). Given the single-center retrospective design, modest sample size, and limited event counts, multivariable modeling was considered exploratory and was not used as the primary basis for inference. A two-sided *P* < 0.05 was considered statistically significant.

### Ethical considerations

The study was approved by the Ethics Committee of The Affiliated Suqian Hospital of Xuzhou Medical University. Because the analysis used de-identified retrospective clinical records and evaluated a routine unit-level nursing practice change, the requirement for additional individual informed consent was waived by the ethics committee. All procedures were conducted in accordance with the Declaration of Helsinki and institutional requirements for confidentiality and clinical data protection.

## Results

### Cohort characteristics

Of 187 consecutive neurosurgical intensive care unit admissions requiring nasogastric EN during the study window, 23 admitted during the implementation transition interval were excluded a priori and 52 did not meet eligibility criteria, yielding 112 patients for analysis: 56 in the usual-care cohort and 56 in the evidence-informed pathway cohort (Figure 1). Baseline demographic and clinical characteristics were balanced between cohorts (Table 1). Men accounted for 30 of 56 patients (53.57%) in the usual-care cohort and 28 of 56 patients (50.00%) in the pathway cohort (χ^2^ = 0.143; *P* = 0.705), and mean age was similar (50.20 ± 10.20 vs. 51.05 ± 10.15 years; *t* = 0.442; *P* = 0.659). The distribution of primary neurosurgical diagnoses did not differ between cohorts (χ^2^ = 0.592; *P* = 0.744); basal ganglia hemorrhage was most common (35 patients, 62.50%, versus 31 patients, 55.36%), followed by ruptured intracranial aneurysm with hemorrhage (15, 26.79%, vs. 18, 32.14%) and massive cerebral infarction (6, 10.71%, vs. 7, 12.50%). Neurologic and overall illness severity were comparable, with similar GCS scores (4.85 ± 1.01 vs. 4.89 ± 1.00; *t* = 0.211; *P* = 0.834) and APACHE II scores (22.63 ± 4.25 vs. 22.68 ± 4.00; *t* = 0.064; *P* = 0.949). Concomitant therapies and hemodynamic support that could influence gastrointestinal motility or splanchnic perfusion were also balanced between cohorts (Table 1). Mechanical ventilation (48 of 56, 85.71%, vs. 47 of 56, 83.93%; *P* = 0.790), vasoactive or catecholamine support (22 of 56, 39.29%, vs. 20 of 56, 35.71%; *P* = 0.697), continuous sedation (41 of 56, 73.21%, vs. 42 of 56, 75.00%; *P* = 0.829), stress-ulcer prophylaxis with acid-suppressive agents (54 of 56, 96.43%, vs. 55 of 56, 98.21%; *P* = 1.000), and prokinetic administration during the observation period (18 of 56, 32.14%, vs. 15 of 56, 26.79%; *P* = 0.534) did not differ between the usual-care and pathway cohorts.

**TABLE 1:** Baseline demographic and clinical characteristics of the study cohorts.

Characteristic	Usual-care cohort (*n* = 56)	Pathway cohort (*n* = 56)	χ^2^/*t*	*P*-value
Sex, n (%)			0.143	0.705
Male	30 (53.57)	28 (50.00)		
Female	26 (46.43)	28 (50.00)		
Age, years, mean ± SD	50.20 ± 10.20	51.05 ± 10.15	0.442	0.659
Primary diagnosis, n (%)			0.592	0.744
Massive cerebral infarction	6 (10.71)	7 (12.50)		
Basal ganglia hemorrhage	35 (62.50)	31 (55.36)		
Aneurysm rupture with hemorrhage	15 (26.79)	18 (32.14)		
GCS score, mean ± SD	4.85 ± 1.01	4.89 ± 1.00	0.211	0.834
APACHE II score, mean ± SD	22.63 ± 4.25	22.68 ± 4.00	0.064	0.949
Mechanical ventilation, n (%)	48 (85.71)	47 (83.93)	0.071	0.790
Vasoactive or catecholamine support, n (%)	22 (39.29)	20 (35.71)	0.152	0.697
Continuous sedation, n (%)	41 (73.21)	42 (75.00)	0.047	0.829
Stress-ulcer prophylaxis, n (%)	54 (96.43)	55 (98.21)	–	1.000
Prokinetic agent use, n (%)	18 (32.14)	15 (26.79)	0.386	0.534

SD, standard deviation; GCS, Glasgow Coma Scale; APACHE II, Acute Physiology and Chronic Health Evaluation II. Categorical variables were compared using the χ^2^ test and continuous variables using independent-samples *t*-tests. Concomitant therapies and hemodynamic support were ascertained over the 14-day observation period. Mechanical ventilation denotes invasive ventilatory support at any time during observation; vasoactive or catecholamine support denotes administration of any vasopressor or inotropic catecholamine; continuous sedation denotes a continuous sedative infusion; stress-ulcer prophylaxis denotes administration of an acid-suppressive agent; and prokinetic agent use denotes administration of a gastrointestinal prokinetic. Cells with an expected frequency below five were compared using the Fisher exact test, for which a test statistic is not reported (denoted by a dash). All *P*-values are two-sided.

### Pathway adherence

Adherence to the evidence-informed pathway in the post-implementation cohort was high. Documented completion was 96.4% for structured prefeeding risk assessment, 98.2% for standardized tube securement and verification, 92.9% for head-of-bed elevation to 30–45 degrees, 94.6% for scheduled tolerance surveillance, and 91.1% for the days 3–7 feeding-compliance review, and the predefined intolerance algorithm was applied in every instance in which an intolerance trigger was documented. The composite measure, reflecting completion of all core elements for an individual patient, was 87.5% (49 of 56), and the mean element-level adherence was 94.6%. Element-by-element adherence is detailed in Supplementary Table S2. These data indicate that the observed associations were achieved under conditions of generally faithful implementation rather than partial uptake.

### Gastrointestinal recovery

**FIGURE 2:**
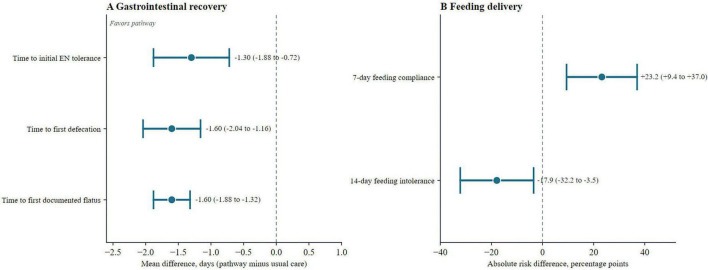
Forest plot of effect estimates with 95% confidence intervals. **(A)** Continuous gastrointestinal recovery outcomes expressed as mean differences in days (pathway minus usual care); negative values favor the pathway. **(B)** Categorical feeding-delivery outcomes expressed as absolute risk differences in percentage points. Confidence intervals complement the point estimates reported in Tables 2, 3. Filled circles denote the point estimate and horizontal bars the 95% CI; the vertical dashed line marks the null value of zero. In panel A, more negative mean differences (days, pathway minus usual care) favor the pathway, as indicated by the “Favors pathway” annotation. In **(B)**, a positive risk difference for 7-day feeding compliance and a negative risk difference for 14-day feeding intolerance both favor the pathway. Continuous outcomes were compared using independent-samples *t*-tests and categorical outcomes using the χ^2^ test, with continuity correction applied to feeding intolerance. CI, confidence interval; EN, enteral nutrition.

The pathway cohort had faster documented gastrointestinal recovery across all three primary endpoints (Figure 2A and Table 2). Time to initial EN tolerance was shorter in the pathway cohort than in the usual-care cohort (6.1 ± 1.5 vs. 7.4 ± 1.6 days; mean difference, −1.30 days; 95% CI, −1.88 to −0.72; *t* = 4.44; *P* < 0.001). Time to first clinically documented flatus was also shorter (2.2 ± 0.7 versus 3.8 ± 0.8 days; mean difference, −1.60 days; 95% CI, −1.88 to −1.32; *t* = 11.26; *P* < 0.001), as was time to first defecation (4.1 ± 0.7 versus 5.7 ± 1.5 days; mean difference, −1.60 days; 95% CI, −2.04 to −1.16; *t* = 7.23; *P* < 0.001). The forest plot in Figure 2A displays these effect estimates with their confidence intervals and indicates a consistent direction of benefit favoring the pathway.

**TABLE 2:** Gastrointestinal recovery outcomes after nasogastric tube placement.

Outcome	Usual-care cohort (*n* = 56)	Pathway cohort (*n* = 56)	Mean difference (95% CI)	*P*-value
Time to initial EN tolerance, days	7.4 ± 1.6	6.1 ± 1.5	−1.30 (−1.88 to −0.72)	< 0.001
Time to first documented flatus, days	3.8 ± 0.8	2.2 ± 0.7	−1.60 (−1.88 to −1.32)	< 0.001
Time to first defecation, days	5.7 ± 1.5	4.1 ± 0.7	−1.60 (−2.04 to −1.16)	< 0.001

Values are mean ± standard deviation. Negative mean differences favor the evidence-informed pathway cohort. CI, confidence interval; EN, enteral nutrition. Mean differences (pathway minus usual care) and their 95% CIs were derived from independent-samples *t*-tests (110 degrees of freedom for each comparison). Initial EN tolerance was defined as continuous nasogastric EN at or above 30 mL per hour sustained for 24 h without intolerance. All reported *P*-values are two-sided, with a threshold of 0.05. SD, standard deviation.

### Feeding compliance and intolerance

Seven-day feeding compliance was higher after pathway implementation (Figure 2B and Table 3). In the usual-care cohort, 39 of 56 patients (69.64%) achieved at least 90% of the prescribed feeding volume by day 7, compared with 52 of 56 patients (92.86%) in the pathway cohort, an absolute increase of 23.2 percentage points (95% CI, 9.4–37.0; χ^2^ = 9.905; *P* = 0.002). Any feeding intolerance during the 14-day observation period was less frequent in the pathway cohort (6 of 56, 10.71%) than in the usual-care cohort (16 of 56, 28.57%), an absolute risk reduction of 17.9 percentage points (95% CI, −32.2 to −3.5; χ^2^ with continuity correction = 4.58; *P* = 0.032; Table 3). Individual manifestations, reported descriptively because of low event counts, included gastric retention (6, 10.71%, vs. 2, 3.57%), nausea or vomiting (5, 8.93%, vs. 2, 3.57%), aspiration (2, 3.57%, vs. 1, 1.79%), diarrhea (1, 1.79%, in each cohort), and abdominal distension (2, 3.57%, v .0). The pathway was therefore associated with both improved achievement of feeding targets and fewer documented intolerance events.

**TABLE 3:** Feeding delivery outcomes during the 14-day observation period.

Outcome	Usual-care cohort (*n* = 56)	Pathway cohort (*n* = 56)	Effect estimate (95% CI)	*P*-value
7-day feeding compliance, n (%)	39 (69.64)	52 (92.86)	+23.2 (9.4–37.0)	0.002
Any feeding intolerance, n (%)	16 (28.57)	6 (10.71)	−17.9 (−32.2 to −3.5)	0.032
Gastric retention, n (%)	6 (10.71)	2 (3.57)	–7.1	–
Nausea or vomiting, n (%)	5 (8.93)	2 (3.57)	–5.4	–
Aspiration, n (%)	2 (3.57)	1 (1.79)	–1.8	–
Diarrhea, n (%)	1 (1.79)	1 (1.79)	0.0	–
Abdominal distension, n (%)	2 (3.57)	0 (0.00)	–3.6	–
Feeding tolerance (no intolerance), n (%)	40 (71.43)	50 (89.29)	+17.9 (3.5 to 32.2)	0.032

Values are n (%) unless otherwise indicated. Seven-day feeding compliance was defined as delivery of at least 90% of the individualized prescribed enteral nutrition volume by day 7. Any feeding intolerance denotes the occurrence of one or more intolerance manifestations during the 14-day observation period; the indented categories are mutually exclusive component events and are reported descriptively because of low event counts. Effect estimates are absolute risk differences in percentage points (pathway minus usual care) with 95% CIs; negative values favor the evidence-informed pathway cohort. *P*-values for 7-day compliance and for overall tolerance status were derived from the χ^2^ test (with continuity correction for tolerance status). CI, confidence interval; EN, enteral nutrition; pp, percentage points.

### Nutritional biomarker trajectories

**FIGURE 3:**
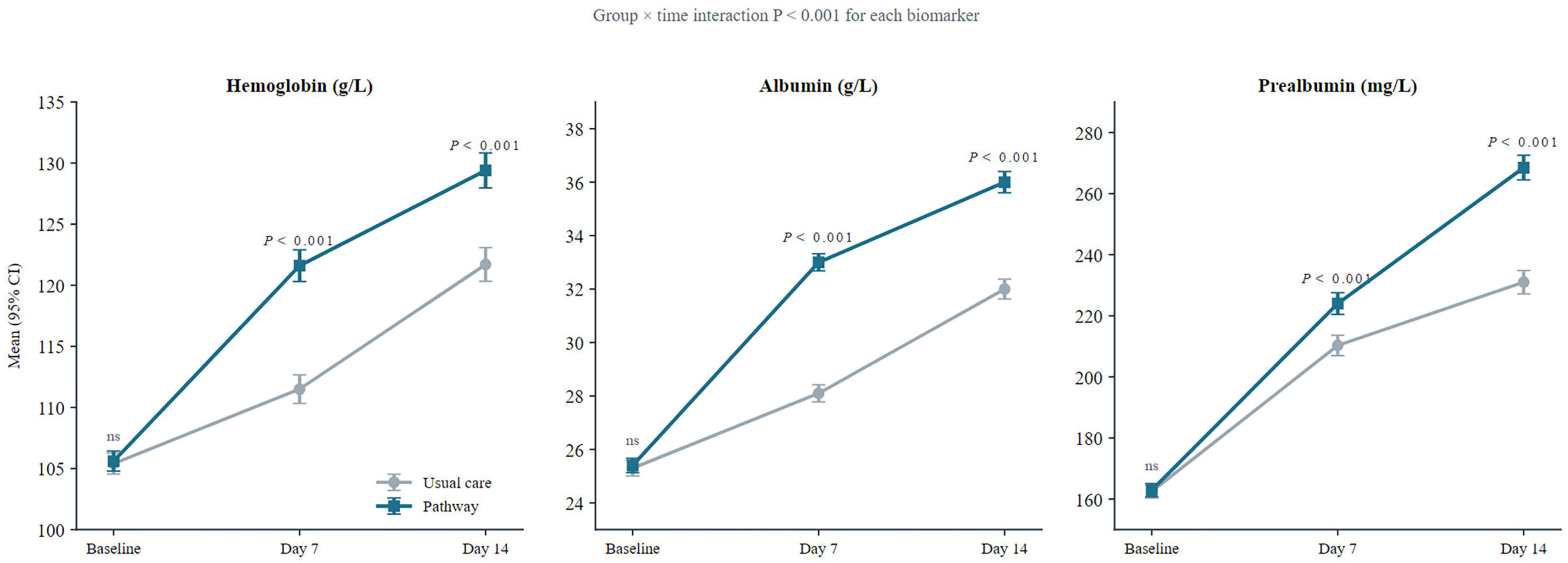
Nutritional biomarker trajectories for hemoglobin, albumin, and prealbumin at baseline, day 7, and day 14 after nasogastric tube placement, by cohort. Markers show the cohort mean and error bars the 95% confidence interval of the mean. The group-by-time interaction was significant for each biomarker (*P* < 0.001), illustrating the widening between-cohort separation over time that the tabulated values alone do not convey. Filled squares denote the evidence-informed pathway cohort and gray diamonds the usual-care cohort. Repeated measurements were analyzed using repeated-measures analysis of variance with group, time, and group-by-time interaction terms and Bonferroni-adjusted pairwise comparisons; the *P*-values displayed at each post-baseline time point are between-cohort comparisons, and “ns” indicates a nonsignificant baseline difference. Hemoglobin and albumin are expressed in g/L and prealbumin in mg/L. CI, confidence interval.

Baseline nutritional biomarkers were comparable between cohorts (Table 4). Hemoglobin, albumin, and prealbumin increased over time in both cohorts, but the increases were greater in the pathway cohort, with significant group-by-time interactions for each biomarker (*P* < 0.001; Figure 3). Hemoglobin rose from 105.6 ± 3.1 g/L at baseline to 121.6 ± 4.9 g/L on day 7 and 129.4 ± 5.4 g/L on day 14 in the pathway cohort, versus 105.4 ± 3.2, 111.5 ± 4.4, and 121.7 ± 5.2 g/L in the usual-care cohort, with between-cohort differences of 10.1 g/L (95% CI, 8.4–11.8) at day 7 and 7.7 g/L (95% CI, 5.7–9.7) at day 14 (both *P* < 0.001). Albumin increased more in the pathway cohort, with between-cohort differences of 4.9 g/L (95% CI, 4.5–5.3) at day 7 and 4.0 g/L (95% CI, 3.5–4.5) at day 14, and prealbumin likewise increased more, with differences of 13.7 mg/L (95% CI, 8.8–18.6) at day 7 and 37.5 mg/L (95% CI, 31.9–43.1) at day 14 (all *P* < 0.001). Triglycerides and total cholesterol remained clinically stable over the 14-day period, with no meaningful between-cohort differences. The widening separation between cohorts over time, shown in Figure 3, is consistent with more reliable nutrition delivery in the pathway cohort, although these biochemical markers are interpreted as supportive rather than definitive.

**TABLE 4:** Nutritional biomarker trajectories before and after nasogastric tube placement.

Biomarker and time point	Usual-care cohort (*n* = 56)	Pathway cohort (*n* = 56)	Between-cohort *P*-value
Hemoglobin, g/L			
Baseline	105.4 ± 3.2	105.6 ± 3.1	0.738
Day 7	111.5 ± 4.4	121.6 ± 4.9	< 0.001
Day 14	121.7 ± 5.2	129.4 ± 5.4	< 0.001
Albumin, g/L			
Baseline	25.3 ± 1.1	25.4 ± 1.0	0.616
Day 7	28.1 ± 1.2	33.0 ± 1.2	< 0.001
Day 14	32.0 ± 1.4	36.0 ± 1.5	< 0.001
Prealbumin, mg/L			
Baseline	162.5 ± 8.0	162.9 ± 8.2	0.794
Day 7	210.3 ± 12.6	224.0 ± 13.4	< 0.001
Day 14	231.0 ± 14.5	268.5 ± 15.2	< 0.001
Triglycerides, mmol/L			
Baseline	2.06 ± 0.05	2.05 ± 0.05	> 0.05
Day 7	2.10 ± 0.05	2.09 ± 0.05	> 0.05
Day 14	2.10 ± 0.05	2.11 ± 0.06	> 0.05
Total cholesterol, mmol/L			
Baseline	4.61 ± 0.05	4.63 ± 0.06	> 0.05
Day 7	4.65 ± 0.05	4.66 ± 0.06	> 0.05
Day 14	4.55 ± 0.06	4.64 ± 0.06	> 0.05

Values are mean ± standard deviation. Repeated measurements were analyzed using repeated-measures analysis of variance; the group-by-time interaction was significant for hemoglobin, albumin, and prealbumin (*P* < 0.001). Biomarkers were measured at baseline, day 7, and day 14 after nasogastric tube placement. Between-group comparisons at each time point were evaluated with Bonferroni-adjusted contrasts following a significant interaction, and the timewise annotations in Figure 3 (ns denoting a nonsignificant baseline difference) correspond to these contrasts. Hemoglobin and albumin are expressed in grams per liter (g/L) and prealbumin in milligrams per liter (mg/L). ANOVA, analysis of variance; SD, standard deviation.

## Discussion

This retrospective quasi-experimental cohort study found that implementation of an evidence-informed nasogastric enteral nutrition nursing pathway was associated with more favorable early nutrition-delivery and gastrointestinal outcomes in critically ill neurosurgical patients. Relative to usual care, the pathway cohort demonstrated shorter time to initial enteral nutrition tolerance, earlier documented flatus and defecation, higher 7-day feeding compliance, fewer 14-day feeding intolerance events, and greater increases in hemoglobin, albumin, and prealbumin during the first 14 days after tube placement. Because the study was nonrandomized and used a before-and-after structure, these findings should be interpreted as associations rather than evidence of causation. Nonetheless, the consistency of effect direction across gastrointestinal recovery, feeding delivery, intolerance, and supportive biochemical markers suggests that standardized nursing procedures may improve the reliability of nasogastric enteral nutrition implementation in this population. The findings are clinically plausible. Neurocritical care patients commonly have reduced consciousness, impaired swallowing, delayed gastric emptying, sedation exposure, procedure-related feeding interruptions, and aspiration risk, all of which render enteral delivery vulnerable to both physiological intolerance and operational inconsistency. Contemporary critical-care nutrition guidance favors enteral over parenteral nutrition when the gastrointestinal tract is functional, while emphasizing gradual advancement and avoidance of overfeeding during early critical illness (1–3). The evaluated pathway translated these principles into a bedside workflow that linked prefeeding risk assessment, tube verification and securement, head-of-bed positioning, progressive rate advancement, predefined intolerance criteria, structured documentation, and escalation. Its novelty is therefore operational rather than nutritional: the pathway introduced no new formula or device but reduced variation in the routine nursing behaviors that determine whether prescribed nutrition is delivered safely and consistently. The reduction in feeding intolerance is particularly important because intolerance frequently precipitates prolonged feeding holds, delayed advancement, and cumulative macronutrient deficits, and its definitions and mechanisms increasingly support proactive surveillance rather than reactive interruption (9). Feeding intolerance is common in general intensive care and is associated with identifiable clinical risk factors, underscoring the value of systematic detection (12). By coupling gradual escalation with predefined responses to gastric retention, vomiting, aspiration concern, diarrhea, and abdominal distension, the pathway may have prevented unnecessary interruption while promoting earlier corrective action. The higher 7-day compliance rate supports this interpretation, because improved target achievement is unlikely if intolerance is overlooked. Consistent with this interpretation, documented adherence to the core pathway elements was high, with a composite adherence of 87.5% and a mean element-level adherence of 94.6% (Supplementary Table S2), indicating that the observed associations arose under conditions of generally faithful implementation rather than partial uptake and strengthening the link between the standardized nursing behaviors and the improvements in feeding delivery. Future iterations might incorporate objective bedside monitoring, such as gastrointestinal ultrasound combined with structured injury scoring, to further individualize advancement (13), and clarify the role of supplemental parenteral nutrition when enteral targets cannot be met in neurosurgical practice (14). The biomarker findings are encouraging but require cautious interpretation. Hemoglobin, albumin, and prealbumin improved more in the pathway cohort, consistent with better feeding delivery; however, albumin and prealbumin are negative acute-phase reactants influenced by inflammation, capillary leak, hydration, hepatic synthesis, renal loss, and exogenous albumin administration, and should not be regarded as stand-alone measures of nutritional repletion (11). Their value here is supportive rather than definitive, particularly because the changes occurred alongside higher feeding compliance and lower intolerance. The magnitude and rapidity of the albumin and prealbumin increases likely reflect resolving acute-phase physiology and concurrent clinical management in addition to nutrition, and this interpretive caution is integral to the conclusions. This study has several limitations. First, the before-and-after design is vulnerable to secular trends, undocumented practice changes, and residual confounding; although baseline age, sex, diagnosis, Glasgow Coma Scale score, and Acute Physiology and Chronic Health Evaluation II score were balanced, unmeasured factors such as sedation depth, vasopressor exposure, ventilation duration, glycemic control, infection, albumin infusion, and procedure-related fasting may have influenced outcomes. The concomitant therapies and hemodynamic support most likely to affect gastrointestinal motility and splanchnic perfusion, including mechanical ventilation, vasoactive or catecholamine support, continuous sedation, acid-suppressive prophylaxis, and prokinetic use, were measured and were comparably distributed between cohorts, which mitigates but does not eliminate the possibility of residual confounding by these factors. Second, the single-center design and modest sample size limit generalizability and precluded stable multivariable adjustment for infrequent intolerance events. Third, bedside staff could not be blinded because the pathway was embedded in routine practice, and documentation intensity may have increased after implementation. Fourth, first flatus is difficult to ascertain precisely in patients with severe impairment of consciousness and should be regarded as a pragmatic documentation endpoint rather than an exact physiological time point. More broadly, both first flatus and first defecation are motility-dependent endpoints that can be modified by sedation, fluid balance, mechanical ventilation, concomitant gastrointestinal-active medications, and the composition of the enteral formula; accordingly, these measures are best interpreted as supportive indicators of gastrointestinal recovery rather than as effects attributable to the nursing pathway in isolation, and the prespecified delivery endpoint of 7-day feeding compliance provides a more directly actionable measure of nutrition delivery. Fifth, downstream patient-centered outcomes, including ventilator-associated pneumonia, infectious complications, length of stay, mortality, and long-term neurologic recovery, were not evaluated, although such outcomes are central to the long-term burden of severe neurologic injury (15) and to comprehensive surveillance of gastrointestinal complications during critical illness (16). In particular, we did not assess aspiration pneumonia, the timing of ventilator weaning, or the timing of transition from enteral to oral feeding, each of which is clinically consequential and plausibly modifiable by more reliable feeding delivery; prospective evaluation of these patient-centered endpoints, together with side-effect reduction and longer-term neurologic prognosis, is a priority for future work. Despite these limitations, the study provides a clinically relevant signal that nursing-led standardization may improve nasogastric enteral nutrition delivery in neurosurgical intensive care. The pathway is reproducible because it depends on structured assessment, feeding advancement, tube safety, intolerance management, and audit rather than specialized equipment. Larger multicenter studies with prospective registration, blinded endpoint adjudication, protocol-fidelity monitoring, and adjustment for severity and treatment variables are needed, and a stepped-wedge or cluster-randomized design would be especially suitable because the intervention is delivered at the nursing-unit level.

## Conclusion

Implementation of a structured evidence-informed nasogastric EN nursing pathway was associated with faster documented gastrointestinal recovery, higher 7-day feeding compliance, fewer feeding intolerance events, and more favorable supportive nutritional biomarker trajectories in critically ill neurosurgical patients. These findings support standardized nursing pathways as a practical strategy for improving the reliability and safety of EN delivery in neurosurgical critical care. Because the study used a retrospective quasi-experimental design, larger multicenter prospective studies are needed to determine whether these improvements translate into better clinical and neurologic outcomes.

## Data Availability

The raw data supporting the conclusions of this article will be made available by the authors, without undue reservation.

## References

[1] Singer P Blaser AR Berger MM Alhazzani W Calder PC Casaer MP et al. ESPEN guideline on clinical nutrition in the intensive care unit. *Clin Nutr*. (2019) 38:48–79. 10.1016/j.clnu.2018.08.037 30348463

[2] Singer P Blaser AR Berger MM Calder PC Casaer M Hiesmayr M et al. ESPEN practical and partially revised guideline: clinical nutrition in the intensive care unit. *Clin Nutr*. (2023) 42:1671–89. 10.1016/j.clnu.2023.07.011 37517372

[3] Compher C Bingham AL McCall M Patel J Rice TW Braunschweig C et al. Guidelines for the provision of nutrition support therapy in the adult critically ill patient: the American society for parenteral and enteral nutrition. *JPEN J Parenter Enteral Nutr*. (2022) 46:12–41. 10.1002/jpen.2267 34784064

[4] Tavarez T Roehl K Koffman L. Nutrition in the neurocritical care unit: a new frontier. *Curr Treat Options Neurol*. (2021) 23:16. 10.1007/s11940-021-00670-8 33814896 PMC8009929

[5] Choi YK Kim HJ Ahn J Ryu JA. Impact of early nutrition and feeding route on clinical outcomes of neurocritically ill patients. *PLoS One*. (2023) 18:e0283593. 10.1371/journal.pone.0283593 36952527 PMC10035931

[6] Barhorst S Prior RM Kanter D. Implementation of a best-practice guideline: early enteral nutrition in a neuroscience intensive care unit. *JPEN J Parenter Enteral Nutr*. (2023) 47:87–91. 10.1002/jpen.2411 35616290 PMC10084295

[7] Hunt CE Kemper C Pauley R Rempel G Visscher D Northington L et al. Reducing the risk of nasogastric tube misplacement: nurse leader responsibility in implementing evidence-based practice. *Nurs Manage*. (2023) 54:34–41. 10.1097/nmg.0000000000000059 37772898

[8] McAuliffe S Archer A Carter A Ray S. An evaluation of nasogastric (NG) tube removal practices and nutritional intake parameters in an acute neurosurgical population: the development of an NG transition feeding protocol. *J Hum Nutr Diet*. (2024) 37:246–55. 10.1111/jhn.13251 37867393

[9] Reintam Blaser A Deane AM Preiser JC Arabi YM Jakob SM. Enteral feeding intolerance: updates in definitions and pathophysiology. *Nutr Clin Pract*. (2021) 36:40–9. 10.1002/ncp.10599 33242218

[10] von Elm E Altman DG Egger M Pocock SJ Gøtzsche PC Vandenbroucke JP. The strengthening the reporting of observational studies in epidemiology (STROBE) statement: guidelines for reporting observational studies. *Lancet*. (2007) 370:1453–7. 10.1016/S0140-6736(07)61602-X 18064739

[11] Evans DC Corkins MR Malone A Miller S Mogensen KM Guenter P et al. The use of visceral proteins as nutrition markers: an ASPEN position paper. *Nutr Clin Pract*. (2021) 36:22–8. 10.1002/ncp.10588 33125793

[12] Yu K Guo N Zhang D Xia Y Meng Y Weng L et al. Prevalence and risk factors of enteral nutrition intolerance in intensive care unit patients: a retrospective study. *Chin Med J*. (2022) 135:1814–20. 10.1097/CM9.0000000000001974 35833658 PMC9521784

[13] Lai J Chen S Chen L Huang D Lin J Zheng Q. Bedside gastrointestinal ultrasound combined with acute gastrointestinal injury score to guide enteral nutrition therapy in critically patients. *BMC Anesthesiol*. (2022) 22:231. 10.1186/s12871-022-01772-9 35854215 PMC9295482

[14] Huang M Yang S Gu A Xu M Sha C. Clinical nursing application of parenteral nutrition combined with enteral nutrition support in neurosurgery. *Afr Health Sci*. (2023) 23:554–60. 10.4314/ahs.v23i3.64 38357139 PMC10862637

[15] Domensino AF Tas J Donners B Kooyman J van der Horst ICC Haeren R et al. Long-term follow-up of critically ill patients with traumatic brain injury: from intensive care parameters to patient and caregiver-reported outcome. *J Neurotrauma*. (2024) 41:123–34. 10.1089/neu.2022.0474 37265152

[16] Alves de Paula J Rabito EI Justino SR Leite LS Dantas D Makiyama da Silva JS et al. Administration of enteral nutrition and gastrointestinal complications in covid-19 critical patients in prone position. *Clin Nutr Open Sci.* (2022) 45:80–90. 10.1016/j.nutos.2022.08.003 36059438 PMC9420200

