# Supplementary material for: Association of an evidence-informed nasogastric enteral nutrition nursing pathway with gastrointestinal recovery and nutritional status in neurosurgical critical care: a retrospective quasi-experimental cohort study
Source: Front Nutr. 2026 Jul 20;13:1883367. doi: 10.3389/fnut.2026.1883367 (PMC13431074; doi:10.3389/fnut.2026.1883367)
Supplement: Supplementary file 1 [file Table_1.DOCX]

**Table S1. Summary of usual-care and evidence-informed pathway elements.**

| **Study phase** | **Usual-care cohort** | **Evidence-informed pathway cohort** |
| --- | --- | --- |
| Prefeeding assessment | Routine clinical assessment documented per existing institutional practice. | Structured aspiration-risk, gastrointestinal-status, tube-safety, and nutrition-risk assessment documented before feeding. |
| Tube placement and verification | Inserted and verified per routine institutional policy. | Inserted, secured, and verified using a standardized checklist with reassessment after suspected displacement. |
| Feeding initiation | Approximately 25 mL/hour when ordered, advanced by bedside judgment and physician direction. | Low-rate initiation with predefined advancement criteria and intolerance-triggered adjustment. |
| Monitoring, first 48 hours | Routine nursing observation and vital-sign monitoring. | Scheduled tube-security checks, head-of-bed assessment, tolerance review, and documentation of interruptions. |
| Days 3 to 7 | Continued routine care and advancement as tolerated. | Progressive advancement toward target with feeding-compliance review and escalation for repeated interruption. |
| Days 8 to 14 | Routine follow-up. | Continued pathway monitoring, intolerance management, and compliance auditing. |

*This supplementary table summarizes the practical distinction between usual care and the evidence-informed nasogastric EN nursing pathway.*

**Table S2. Documented adherence to core elements of the evidence-informed nasogastric enteral nutrition nursing pathway in the post-implementation cohort (n = 56).**

| **Pathway element** | **Patients with documented completion** | **Adherence, %** |
| --- | --- | --- |
| Structured prefeeding risk assessment | 54 of 56 | 96.4 |
| Standardized tube securement and verification | 55 of 56 | 98.2 |
| Head-of-bed elevation to 30 to 45 degrees | 52 of 56 | 92.9 |
| Scheduled tolerance surveillance | 53 of 56 | 94.6 |
| Days 3 to 7 feeding-compliance review | 51 of 56 | 91.1 |
| Intolerance algorithm applied when triggered | All triggered instances | 100.0 |
| Composite adherence (all core elements) | 49 of 56 | 87.5 |

*Adherence was ascertained by structured audit of the nursing record for each patient managed under the pathway. Element-level adherence is expressed as the number of patients with documented completion of the element divided by the 56 patients in the pathway cohort. The intolerance algorithm was evaluated only among patients in whom an intolerance trigger was documented and was applied in every such instance. Composite adherence reflects documented completion of all core elements for an individual patient. The mean of the five scheduled element-level adherence rates was 94.6%. EN, enteral nutrition.*
